# Imaging the Impact of Chemically Inducible Proteins on Cellular Dynamics *In Vivo*

**DOI:** 10.1371/journal.pone.0030177

**Published:** 2012-01-19

**Authors:** Hon S. Leong, Michael M. Lizardo, Amber Ablack, Victor A. McPherson, Thomas J. Wandless, Ann F. Chambers, John D. Lewis

**Affiliations:** 1 Translational Prostate Cancer Research Group, London Regional Cancer Program, London, Ontario, Canada; 2 Department of Medical Biophysics, University of Western Ontario, London, Ontario, Canada; 3 London Regional Cancer Program, London, Ontario, Canada; 4 Department of Chemical and Systems Biology, Stanford University, Stanford, California, United States of America; Stanford University, United States

## Abstract

The analysis of dynamic events in the tumor microenvironment during cancer progression is limited by the complexity of current *in vivo* imaging models. This is coupled with an inability to rapidly modulate and visualize protein activity in real time and to understand the consequence of these perturbations *in vivo*. We developed an intravital imaging approach that allows the rapid induction and subsequent depletion of target protein levels within human cancer xenografts while assessing the impact on cell behavior and morphology in real time. A conditionally stabilized fluorescent E-cadherin chimera was expressed in metastatic breast cancer cells, and the impact of E-cadherin induction and depletion was visualized using real-time confocal microscopy in a xenograft avian embryo model. We demonstrate the assessment of protein localization, cell morphology and migration in cells undergoing epithelial-mesenchymal and mesenchymal-epithelial transitions in breast tumors. This technique allows for precise control over protein activity *in vivo* while permitting the temporal analysis of dynamic biophysical parameters.

## Introduction

Intravital imaging is a powerful tool to define the impact of specific perturbations in target cells in real time, allowing one to test concepts gathered *in vitro* while providing instructive observations not readily captured by the histological evaluation of tissue. In practice, intravital imaging approaches are limited by our inability to: 1) rapidly toggle the expression level of target proteins and; 2) visualize the impact on cellular biophysics *in vivo* over physiologically relevant timeframes (12–48 hrs). While imaging windows such as cranial, dorsal flap and mammary fat pad windows permit intravital imaging of tumors, the requirements for anesthesia, surgery, hydration maintenance and control of breathing motion artifacts during image acquisition must be considered [1], [2], [3]. To circumvent these limitations, we have developed a technique that combines protein-level chemical modulation with a real-time imaging platform to visualize human tumor xenografts in the shell-less chick embryo for extended periods of time. Removing the requirement for invasive procedures, anesthesia or feeding, this approach allows for continuous intravital imaging for 48 hours or more without adverse effects on the host or the cancer cell xenografts. Furthermore, the shell-less configuration provides accessibility to the tumor and surrounding vasculature, which permits the intravenous administration of a chemical inducer during the imaging experiment.

**Figure 1:**
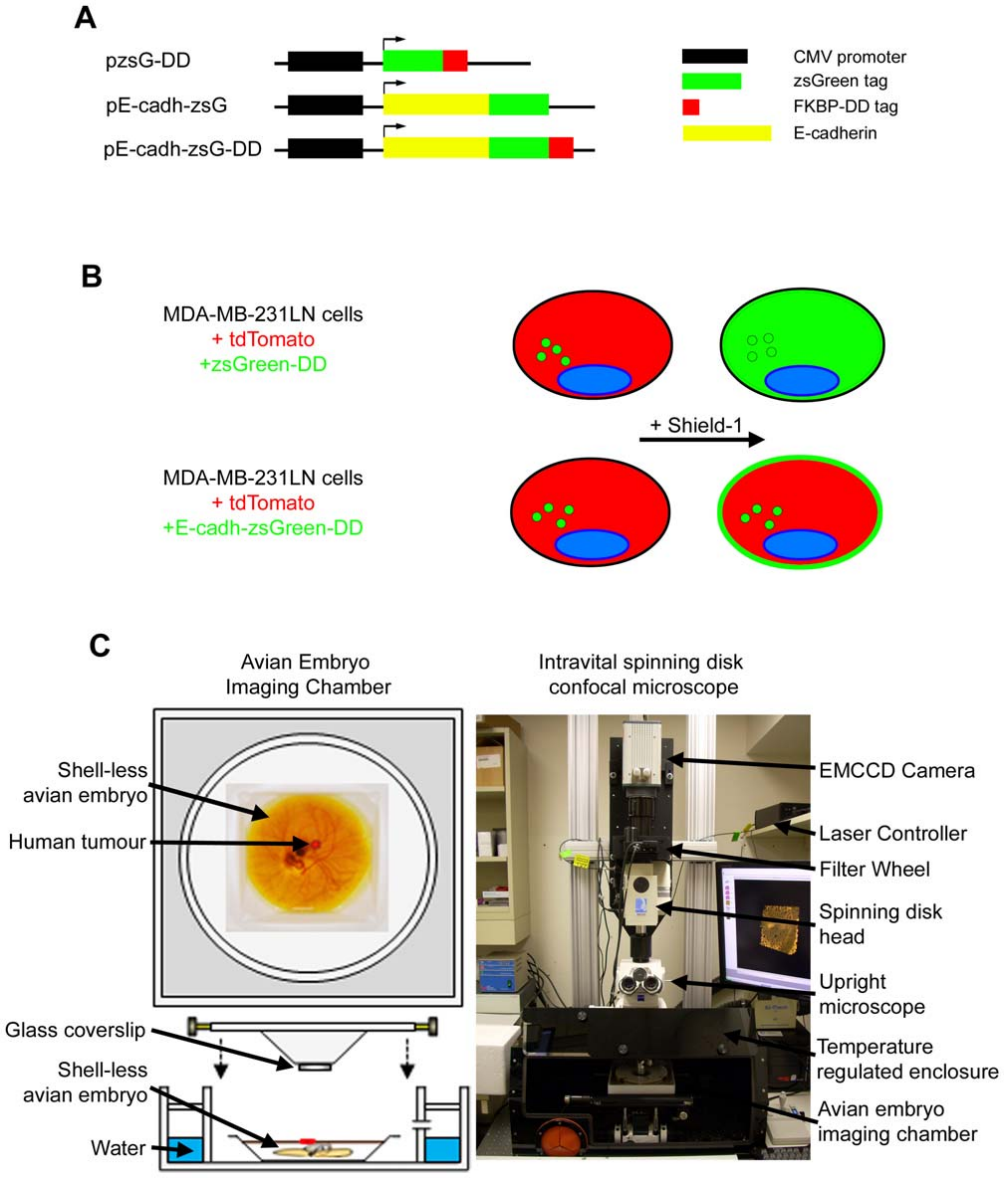
A chemically tunable form of E-cadherin for use in intravital imaging. A) Expression vectors encoding tunable zsGreen (pzsGreen-DD), fluorescent E-cadherin (pE-cadh-zsG) and tunable fluorescent E-cadherin (pE-cadh-zsG-DD). Components include CMV promoter (pCMV), zsGreen fluorescent protein (zsGreen), the Shield-1 binding degradation domain (FKBP-DD), and E-cadherin. B) Schematic of MDA-MB-231-luc-D3H2LN (231LN) cells used to express tunable proteins and the predicted behavior of cells in the presence or absence of Shield-1. 231LN tumor cells were stably transfected with tdTomato and zsGreen alone or as a fusion with E-cadherin. C) Intravital imaging platform (right) with avian embryo imaging chamber (left) to maintain proper temperature (37°C) and humidity (>90%) used to perform *in vivo* three dimensional time-lapse imaging of micrometastases in the chorioallantoic membrane of the avian embryo.

Chemical induction systems based on the Tet-ON/OFF system suffer from lengthy chemical induction lag [4], promoter leakiness and variable doxycycline clearance times [5], [6]. Doxycycline also affects vascular permeability [7] and exhibits anti-angiogenic activity [8], [9] through inhibition of MMP-8, MMP-13 [10] and MMP-9 transcription [11], making it less than ideal for the investigation of the tumor microenvironment. In contrast, the FKBP-destabilization domain (DD) and its chemical inducer, Shield-1, is a chemical induction system that operates at the post-translational level and overcomes many of the limitations associated with transcription-level induction systems. A target protein fused with the FKBP-DD tag is constitutively synthesized but promptly degraded by the cell's proteasome in the absence of the hydrophilic small molecule, Shield-1 [12], [13]. Upon the addition of Shield-1 ligand, the FKBP-DD tag of the target protein is stabilized through a direct binding event, preventing its degradation and rendering the target protein active (Figure 1B) [12], [13]. The induction of protein activity is rapid, resulting in accumulation of target protein within the cell within hours (Figure 1B–C and [12]). Moreover, the depletion of Shield-1 results in an equally rapid transition to protein degradation, which allows the user to “toggle” a target protein on and off during a single continuous intravital imaging experiment.

We applied this imaging and induction technique to visualize epithelial-mesenchymal transitions (EMT) in the MDA-MB-231-luc-D3H2LN human breast carcinoma cell line by chemically modulating intracellular levels of E-cadherin (E-cadh). E-cadherin is a tumor suppressor protein [14], [15] that is important for maintaining cell-to-cell contacts between epithelial cells [16]. The loss of E-cadherin expression is associated with an invasive phenotype as observed in metastatic cancer cell lines [16], [17]. Loss of E-cadherin expression is a hallmark of epithelial-mesenchymal transition (EMT), during which epithelial cells lose many of their epithelial characteristics and acquire certain properties of mesenchymal cells, conferring increased motility and invasiveness. Mounting evidence suggests that cancer cells exhibit a dynamic plasticity between epithelial and mesenchymal states that allows them to survive at distinct steps of metastasis [18], [19], [20], [21]. While the consequence of the manipulation of EMT-related factors has been studied extensively, cycling of EMT has not been directly visualized *in vitro* or *in vivo* in real-time. To achieve this, a chemically inducible form of E-cadherin was introduced into MDA-MB-231-luc-D3H2LN breast cancer cells (henceforth known as 231LN) (Figure 1B), which express negligible levels of endogenous E-cadherin similar to the parental MDA-MB-231 cell line (data not shown) [22]. To determine the impact of this E-cadherin chimera on EMT, we optimized conditions for the rapid induction and depletion of E-cadherin with the objective of visualizing transitions between mesenchymal and epithelial morphological states both *in vitro* and *in vivo*. These transitions were captured in real time using 3D time-lapse videomicroscopy over periods of up to 48 hours.

This technique allows one to directly visualize the impact of target protein modulation on human cells *in vivo* using intravital imaging. The use of standard high resolution microscopy objectives permits the dynamic visualization of target protein subcellular localization, as well as the quantitation of key biophysical information such as cell morphology and migration. Importantly, this methodology is widely applicable to a wide variety of cell types, target proteins, or higher throughput approaches.

## Results

### Concentration-dependent kinetics of Shield-1 mediated protein stabilization

**Figure 2:**
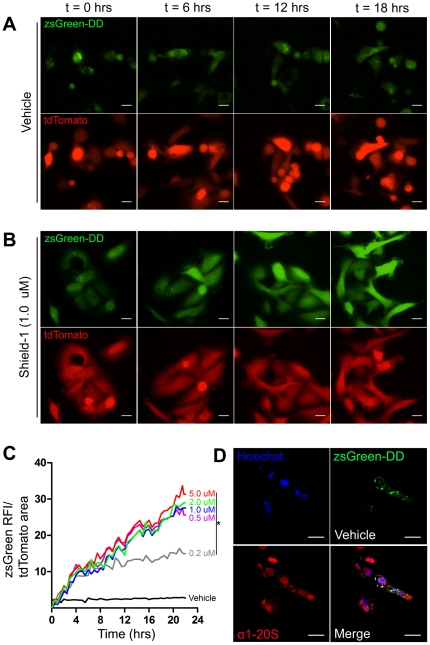
Rapid induction of the fluorescent protein zsGreen in MDA-MB-231LN (231LN) cells *in vitro*. 231LN cells containing both tdTomato and zsGreen-DD were grown on glass coverslips. Panels represent fluorescence time-lapse imaging of 231LN cells treated with vehicle (A) and 1.0 µM of Shield (B). C) Quantification of zsGreen signal within the cells in the presence and absence of Shield-1 over time (*denotes p<0.01 compared to Vehicle treatment kinetic, N>10 cells per field of view, 10 fields of view analyzed per group). Treatment with 0.5, 1.0 and 2.0 µM Shield-1 revealed similar first order kinetics, while treatment with 0.2 µM Shield-1 revealed a similarly steep but brief increase (induction) in signal accumulation followed by a less steep kinetic at 4 hours post-treatment (depletion kinetic). D) Fluorescence immunohistochemistry demonstrates co-localization of proteasome (α1-20S antibody in red) with zsGreen-DD signal in 231LN cells in the absence of Shield-1. All scale bars are 25 µm.

To establish the kinetics of Shield-1-mediated target protein modulation in the 231LN cell line, a vector encoding the green fluorescent zsGreen protein fused in-frame with the DD domain (protein: zsG-DD; vector: pzsG-DD – Figure 1A) was stably introduced (Figure 1B). When these cells were treated with vehicle (media + 1% EtOH), zsG-DD signal localized to perinuclear compartments (Figure 2A) and no increase in total fluorescence was observed over 24 hours (Figure 2C, Movie S1 – top panels). Treatment with 0.5, 1.0, 2.0 and 5.0 µM Shield-1 resulted in an equivalent rapid first-order increase in zsG-DD signal throughout the cell interior (Figure 2B) over 24 hours (Figure 2C, Movie S1 - 1.0 µM treatment, bottom panels). This indicated that maximal induction, presumably limited by the protein expression machinery, was occurring at 0.5 µM Shield-1 and above. Treatment with 0.2 µM Shield-1 resulted in a bi-phasic induction kinetic as evidenced by a lower slope in the induction curve after 4 hours of treatment compared to the Shield-1 treatments >0.5 µM. This indicated that Shield-1 levels were limiting at this concentration after 4 hours of treatment with 0.2 µM and therefore this concentration was utilized for the subsequent E-cadherin induction and depletion experiments. Moreover, the biphasic slopes in the 0.2 µM Shield-1 treatment indicated that zsG-DD protein exhibited minimal turnover and hence a long intracellular half-life, thus validating its use as a reporter of Shield-1 bioavailability. In the absence of Shield-1, zsG-DD protein (green channel) localized with the 20S proteasome subunit (red channel) (Figure 2D) with a Pearson's coefficient of R = 0.73, indicating substantial co-localization within 231LN cells. In addition to establishing the concentration-dependent kinetics of Shield-1 mediated protein stabilization, these experiments suggested that 231LN cells expressing tunable zsG-DD protein would be suitable pharmacokinetic reporters of Shield-1 bioavailability.

### Toggling the accumulation and degradation of E-cadherin to mediate EMT

**Figure 3:**
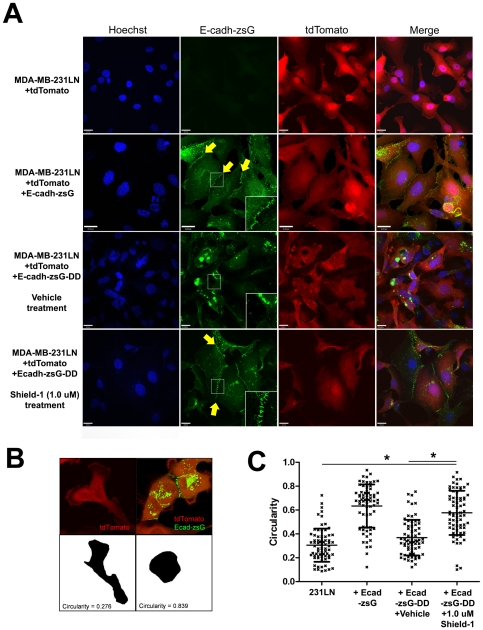
Characterization of tunable E-cadherin-zsG-DD protein expression in 231LN cells *in vitro*. A) Representative images of 231LN cells expressing fluorescent E-cadherin chimeras. Cell nucleus as stained by Hoechst (blue), E-cadherin-zsGreen (green), and tdTomato to highlight the cytoplasm (red) reveal the changes in cell morphology when E-cadherin is over-expressed (row 2) or induced with Shield-1 for 12 hours (row 4) compared to control (row 1) or un-induced cells (row 3). Arrows (yellow) highlight junctions formed by Shield-1-stabilized E-cadh-zG-DD. Scale bars are 20 µm. Insets show magnified view (250%) of cellular junctions. B) Examples of circularity measurements of representative 231LN cells (left) and 231LN cells expressing E-cadh-zsG-DD treated with 1.0 µM Shield-1 (right). C) Circularity measurements to assess a mesenchymal vs. epithelial morphology in cells described above. N = 70 per group, * denotes p<0.01 between groups, 2-way ANOVA.

**Figure 4:**
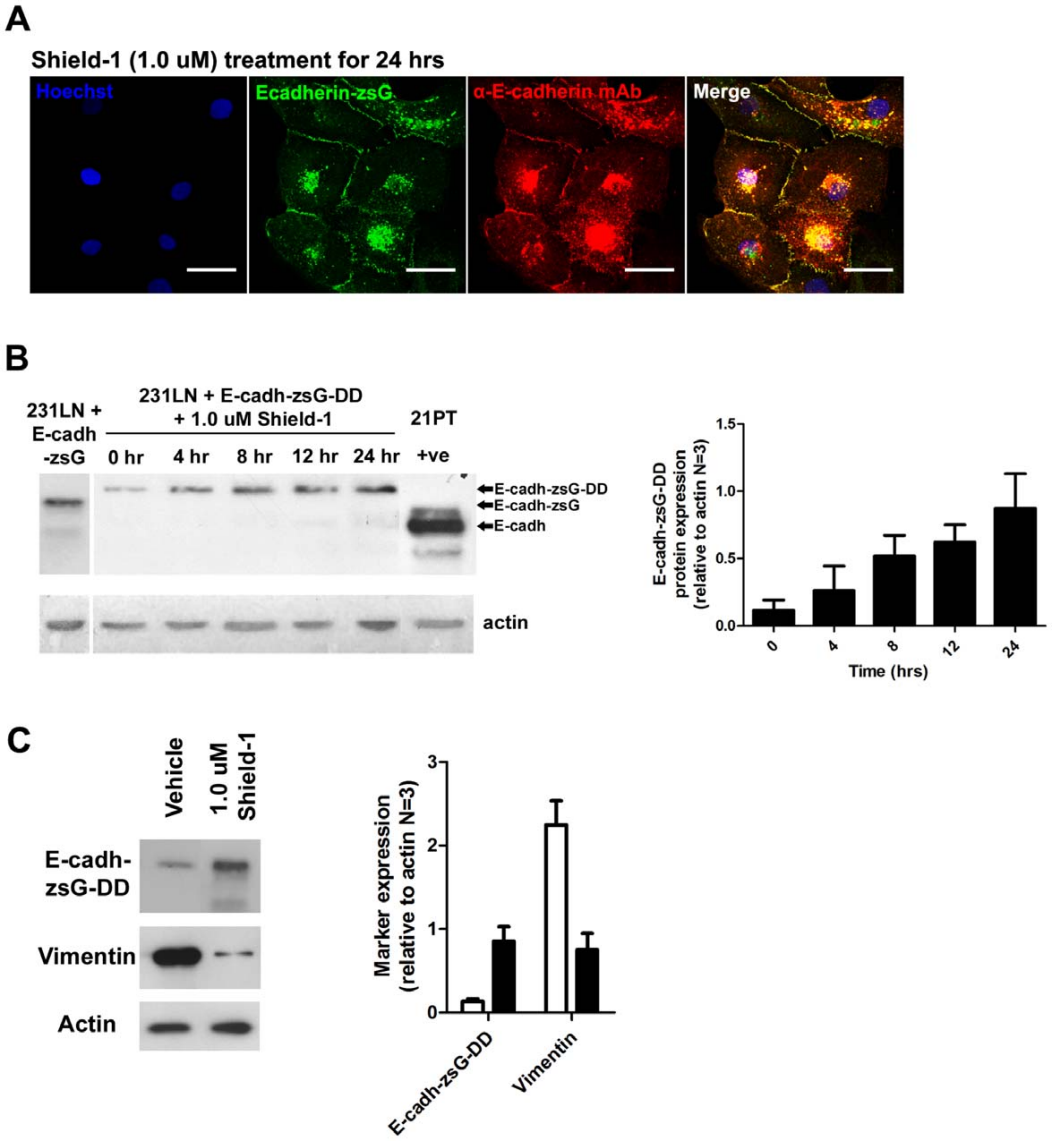
Induction of E-cadherin-zsG-DD protein in 231LN cells by Shield-1 ligand and expression of vimentin. A) 231LN cells expressing E-cadh-zsG-DD (green) treated with 1.0 µM Shield-1 for 24 hours and immunostained with anti-E-cadherin mAb (red) and Hoechst nuclear stain (blue). Scale bars are 25 µm. B) Western immunoblot analysis of E-cadherin expression in 231LN cells expressing E-cadh-zsG-DD and treated with 1.0 µM Shield-1 using the same mAb as in A). Graph (right) represents analyses performed on three independent induction experiments. Cell lysates of 231LN cells expressing E-cadherin-zsG are shown in the first lane. Lysates of cells expressing E-cadherin-zsG-DD were collected at 0, 4, 8, 12, 16, and 24 hrs after Shield-1 treatment (1.0 µM final), revealing accumulation of Shield-1 stabilized E-cadherin-zsG-DD within cells (∼135 kDa). Far right lane is a positive control of 21PT cells [32] which endogenously express high levels of E-cadherin (∼110 kDa). C) Western immunoblot analysis of markers for epithelial-mesenchymal transition (EMT). Blot (left panels) reveals a decrease in vimentin protein levels when E-cadh-zsG-DD is induced by 1.0 µM Shield-1 treatment. Graph (right) represents analyses performed on three independent induction experiments.

E-cadherin plays a pivotal role in the establishment and stabilization of the cell-cell junctions that are characteristic of epithelial cells, and cultured 231LN cells exhibit a spindle-shaped, mesenchymal cell morphology *in vitro* (Figure 3A - top panels) but can assume an epithelial, cobble-stone morphology when E-cadherin is re-expressed [22], [23]. We expected that inducing the accumulation of E-cadherin would result in a morphological transition from a mesenchymal morphology to an epithelial one. To confirm this in our model, E-cadherin was stably expressed in 231LN cells. In contrast to the parental cells (Figure 3A, top panels), stable re-expression of E-cadherin-zsG (E-cadh-zsG) induced the formation of zsG-labeled junctions (arrows) and an epithelial, cobblestone morphology (Figure 3A, second row of panels from the top). Next, the effect of Shield-1 mediated induction of E-cadherin expression was assessed in real time using time-lapse fluorescence microscopy. In the absence of Shield-1, 231LN cells expressing a tunable fluorescent E-cadherin-DD (E-cadh-zsG-DD) continued to exhibit a mesenchymal morphology (Figure 3A, third row of panels from the top), while Shield-1 treated cells rapidly formed cell-cell junctions (arrows) and transitioned to an epithelial morphology (Figure 3, last row of panels). A monoclonal antibody for human E-cadherin confirmed the membrane localization of stabilized E-cadh-zsG-DD in immunostained 231LN cells treated with Shield-1 (Figure 4A), and overall levels of E-cadherin in these cells accumulated over 24 hours as demonstrated by Western blot (Figure 4B). Taken together, these observations validate that the tunable chimera E-cadh-zsG-DD is efficiently induced by Shield-1 and behaves equivalently to re-expressed native E-cadherin.

Induction of E-cadh-zsG-DD expression in 231LN cells resulted in a shape change from a spindle mesenchymal morphology to an polygonal epithelial morphology. To quantitate this change in shape, circularity measurements of individual cells from each group (Figure 3B) was used. Circularity measures the “roundness” of the two-dimensional shape of each cell, hence a perfect circle will exhibit a circularity value of 1.0, an epithelial cell will exhibit a circularity value approximating 1.0, while a spindle-shaped cell will exhibit a circularity value approximating 0.0. For example, 231LN cells in Figure 3B (left panel) exhibit a circularity value of 0.276 because of their elongated spindle shape (Figure 3B, left panel) [24], [25], [26], [27], whereas over-expression of E-cad-zsG in these same cells will induce a shape change to a polygonal epithelial morphology with a circularity value of 0.839 (Figure 3B, right panel). Overall, Shield-1 induction of E-cadh-zsG-DD in 231LN cells induced a shape change to a more rounded morphology (Figure 3C), as evidenced by a significantly higher circularity index value compared to 231LN cells (far left) and vehicle treated cells (second from right) and similar to circularity values exhibited by the 231LN cells expressing E-cadh-zsG (second from left, * denotes p<0.01, one-way ANOVA, N = 70 each group).

Alongside the observed changes in cell shape due to E-cadh-zsG-DD induction, protein levels of EMT markers such as vimentin were also changed with Shield-1 treatment. In the presence of vehicle, cells expressed high levels of vimentin and negligible levels of E-cadh-zsG-DD protein consistent with the mesenchymal phenotype of the 231LN cells. However, treatment with 1.0 µM Shield-1 for 12 hours resulted in an increase in E-cadh-zsG-DD protein and a decrease in vimentin protein levels. The expression levels of these EMT markers according to immunoblot analysis underscores the observed shape change from a mesenchymal to epithelial phenotype upon Shield-1 induction.

**Figure 5:**
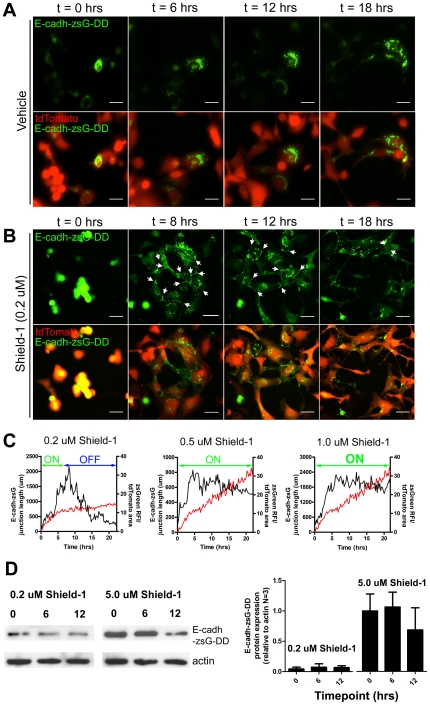
Time-lapse imaging of E-cadherin induction and kinetics of cell-cell junction formation *in vitro*. Fluorescence *in vitro* time-lapse imaging of 231LN cells containing inducible E-cadherin treated with vehicle (A) or 0.2 µM Shield-1 which will produce an induction and depletion effect over 24 hours (B). Data Scale bar is 25 µm. C) Measure of “actively engaged" E-cadherin in 231LN cells in the presence of varying levels of Shield-1 (0.2, 0.5, and 1.0 µM Shield-1), expressed as the cumulative length of all zsG-positive adherens junctions over time (µm/hrs) in representative time-lapse experiments. The black kinetic represents the total cumulative length of E-cadherin-based junctions within a field of view at that timepoint while the red kinetic represents the accumulation of zsGreen-DD exposed to similar Shield-1 treatment. The “induction” and “depletion” phases of chemical induction are annotated in each graph. D) Conditioned media collected from cells expressing pzsGreen-DD which were treated with 0.2 µM Shield for 0, 6 and 12 hours were used to induce E-cadherin-zsGreen-DD expression in 231LN cells expressing E-cad-zsG-DD. There is induction with the 0 and 6 hours conditioned media, but minimal effect with the 12 hour conditioned media. Conversely, conditioned media from cells treated with 5.0 µM Shield-1 induced E-cadherin-zsG-DD expression regardless of the time of conditioned media collection. Graph (Figure 5D, right) represents data from three independent western immunoblot experiments.

Given the rapid kinetics of target protein accumulation and the establishment of a Shield-1-limited dose, we surmised that E-cadherin accumulation and degradation in the 231LN cell line could be "toggled" by the introduction and withdrawal of Shield-1. To assess this, 231LN cells expressing tunable E-cadherin (E-cadh-zsG-DD) were treated with 0.2 µM Shield-1 and visualized over 24 hours using time-lapse fluorescence microscopy. E-cadherin-positive junctions were measured by tracing the outline of each cell, tracing the zsGreen positive junctions where the cells intersect, and then determining the cumulative length using ImageJ. Compared to vehicle treatment (Figure 5A –Movie S2, top panels), 0.2 µM Shield-1 treatment resulted in the formation of E-cadherin-containing junctions between neighboring cells within 4 hours (Movie S2, bottom panels). These junctions remained intact and then declined over the next 8 hours (Figure 5B – Movie S2, bottom panels), coinciding with the Shield-1-limited kinetics observed in cells expressing tunable zsGreen (Figure 2C and Figure 5C left panel). While the 0.5 and 1.0 µM Shield-1 treatments result in a rapid induction effect, Shield-1 depletion was not observed under these ligand concentrations (Figure 5C middle and right panels). To confirm that these observations were indeed due to the depletion of bioavailable Shield-1, conditioned media from tunable zsGreen cells treated with 0.2 µM and 5.0 µM Shield-1 ligand at 0, 6, and 12 hrs post-treatment were used to treat 231LN cells expressing tunable E-cadherin (E-cadh-zsG-DD). Conditioned media from 0.2 µM Shield-1-treated cells collected at 6 and 12 hrs post-induction was not capable of inducing E-cadh-zsG-DD accumulation in cells expressing E-cadh-zsG-DD (Figure 5D), whereas media from earlier timepoints or media from cells treated with 5.0 µM Shield-1 at all timepoints retained the ability to induce E-cadh-zsG-DD in cells expressing E-cad-zsG-DD. These data demonstrate that target proteins such as zsG-DD and E-cadh-zsG-DD can be rapidly induced and then degraded in 231LN cells within 24 hours with predictable kinetics when a 0.2 µM Shield-1 concentration is utilized. Furthermore, when the accumulation of E-cadherin is toggled in this manner in 231LN cells, an inducible but transient epithelial morphology can be observed.

### An *in vivo* pharmacokinetic reporter of Shield-1 bioavailability

**Figure 6:**
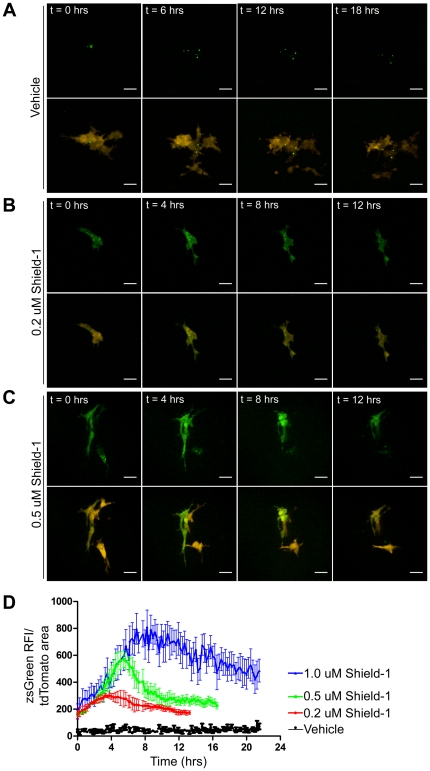
Intravital time-lapse imaging of fluorescent protein induction in 231LN cells *in vivo*. 231LN cells expressing tdTomato (red) and inducible zsGreen-DD (green) were injected intravenously in the avian embryo and allowed to extravasate and proliferate into micrometastases. Representative time-lapse images (maximum intensity projections) are shown after intravenous administration of Vehicle (A), 0.2 µM Shield-1 (B), and 0.5 µM Shield-1 (C). D) Quantification of *in vivo* zsGreen fluorescence in tdTomato-positive cells over time. Data for Vehicle (black kinetic), 0.2 µM Shield-1 (red kinetic), 0.5 µM Shield-1 (green kinetic), and 1.0 µM Shield-1 (blue kinetic) are represented as averages of at least three movies analyzed in each group. Error bars are SE and scale bar represents 25 µm.

To assess the pharmacokinetics of Shield-1 in the shell-less avian embryo xenograft model, 231LN cells expressing tunable zsGreen (zsG-DD) were injected intravenously and allowed to extravasate and form micrometastatic colonies as we have previously described [28]. The response of the tumor cells to the systemic administration of a range of Shield-1 concentrations was assessed *in vivo* using intravital confocal microscopy whereby three dimensional time-lapse movies were acquired at high magnification for 12-18 hours (Figure 6, Movie S3). Micrometastatic colonies of 231LN cells, identified by virtue of their cytoplasmic tdTomato fluorescence, were assessed for changes in zsGreen signal subsequent to intravenous Shield-1 administration (Figure 6D). At final Shield-1 concentrations ranging from an estimated 0.2–1.0 µM , an initial and rapid accumulation of zsG-DD protein was observed in the cytoplasm of 231LN cells (Figure 6A–C) while exhibiting no observable toxic effects on avian embryo viability. The time to attain maximal fluorescence and the degree of maximal fluorescence was Shield-1 concentration-dependent (Figure 6D), and was followed by a gradual reduction in fluorescence (Figure 6D). The estimated 0.5 µM final Shield-1 concentration generated a consistent and reproducible induction of zsG-DD that peaked at 6 hours, followed by a degradation phase that returned to half-maximal fluorescence by 10 hours. These experiments established the utility of 231LN cells expressing the tunable zsGreen protein as a useful *in vivo* reporter for the real-time bioavailability of Shield-1. Furthermore, they confirm the feasibility of performing both induction and degradation phase analyses of target proteins during a single continuous intravital imaging experiment.

### Intravital visualization of reversible mesenchymal to epithelial transitions in cancer cells *in vivo*

**Figure 7:**
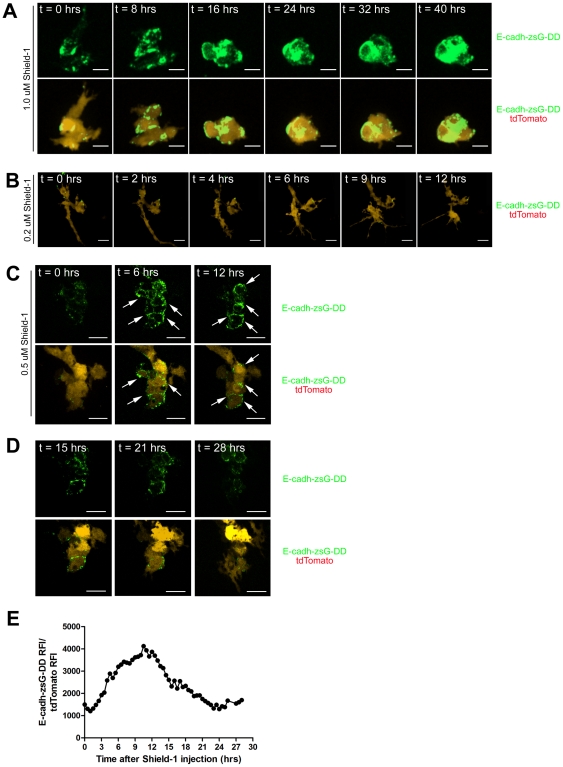
Induction of E-cadherin causes morphological changes in 231LN micrometastases. 231LN cells expressing tdTomato (red) and tunable E-cadherin-zsGreen-DD (green) were injected intravenously in the avian embryo and allowed to extravasate and proliferate into micrometastases. Representative maximum intensity projections are shown. A) *In vivo* treatment with 1.0 µM Shield-1 demonstrates transition from a mesenchymal morphology to an epithelial morphology and continued maintenance of the epithelial morphology over an extended period of time (>40 hrs). Formation of E-cadherin junctions is apparent at t = 0.5 hrs, increasing through 24 hrs. B) Representative micrometastatic colony expressing tunable E-cadherin-zsG-DD and treated with 0.2 µM Shield-1. No induction effect is observed with 0.2 µM Shield-1 *in vivo*. C) Single Z-plane slices of a representative micrometastastic colony expressing tunable E-cadherin-zsG-DD and treated with 0.5 µM Shield-1. These panels represent the stabilization effect induced by 0.5 µM Shield-1 over the 12 hour time course; E-cadh-zsG-DD is stabilized and junctions appear between 231LN cells. E-cadh-zsG-DD junctions between cells of the micrometastatic colony are highlighted by arrows. D) Panels represent the depletion effect in the same colony with depleted levels of 0.5 µM Shield-1; E-cadh-zsG-DD junctions gradually disperse over time and 231LN cells eventually revert to a mesenchymal morphology. All scale bars are 25 µm. E) Quantitation of E-cadh-zsG-DD signal in 231LN-tdTomato cells in a 4-dimension image set over the entire 28 hour time course.

Proof of principle experiments were conducted to assess the impact of sustained and transient expression of E-cadherin in micrometastatic 231LN tumor cell colonies. For the sustained induction experiments, a 1.0 µM Shield-1 final concentration *in vivo* was selected as this generated a persistent induction stimulus. Micrometastatic colonies were visualized using intravital confocal imaging over a period of 40 hours. When a 1.0 µM Shield-1 concentration was utilized, 231LN tumor cell colonies rapidly transitioned from an invasive mesenchymal morphology to a tightly packed "globular" epithelial morphology (Figure 7A, Movie S4). Increasingly stable cell-cell junctions were clearly observed between tumor cells, highlighted by the Shield-1 stabilized fluorescent E-cadh-zsG-DD protein. The intravenous administration of 0.2 µM Shield-1 did not result in any E-cadherin induction *in vivo* (Figure 7B, Movie S5) and therefore Shield-1 final concentrations of 0.5 µM and above were used for visualizing transitions between the mesenchymal and epithelial cell states.

These experiments clearly illustrate that sustained E-cadherin expression is sufficient to alter the morphology and behavior of 231LN cells *in vivo*. To test whether the observed changes would persist after a subsequent destabilization of E-cadh-zsG-DD, chick embryos bearing 231LN micrometastases were treated with 0.5 µM Shield-1. Again, induction of E-cadherin resulted in a rapid morphological transition to an epithelial phenotype during the initial 6 hours, concurrent with an accumulation of fluorescent E-cadherin chimera at the tumor cell junctions (Figure 7C, Movie S6). The morphological transition is particularly dramatic when compared to a cell in the same field that does not express the E-cadherin chimera, exhibiting a highly invasive morphology. This cell does not exhibit E-cadh-zsG-DD signal over the entire time course as revealed in the single Z-plane image set presented in Figure 7C and D. This dynamic morphological effect caused by Shield-1 induction is further conveyed upon comparison to other tdTomato-positive cells within the field of view, which do not assume an epithelial morphology throughout the entire time course. After 15 hours, E-cadh-zsG-DD dissociates from the cell junctions and the cells revert back to their spindle-shaped, mesenchymal morphology (Figure 7D, Movie S7). The localization of E-cadh-zsG-DD at t = 28 hours is comparable to that at t = 0 hours, confirming that the degradation of the E-cadherin chimera has resumed. Of note, is that the initial mesenchymal morphology at t = 0 is not identical to the final mesenchymal morphology at t = 28 due to cell proliferation, migration and E-cadh-zsG-DD induction. These experiments demonstrate that consistent and sustained levels of E-cadh-zsG-DD are required to maintain an epithelial morphology in 231LN cells and that the destabilization of E-cadherin results in a rapid reversion to a more mesenchymal and invasive morphology.

## Discussion

This technique is particularly useful for the visualization of dynamic transitions in cellular morphology in response to the rapid induction and/or degradation of a transgenic protein, such as those that mediate EMT. The coupling of a responsive chemical induction system with a long term *in vivo* imaging platform allows for a precise linkage between the level of target protein with the collection of both qualitative morphological and quantitative biophysical information. Given the technical challenges of continuous real-time intravital imaging of subcellular structures, the timing of induction and knowledge of any prolonged effect is critical in the interpretation of cell biophysics, such as during chemically-induced perturbations of cell function. Cell biophysical parameters such as morphology, cell protrusion formation and migration can be simultaneously assessed during Shield-1 mediated induction of a target protein, while avoiding the induction lag suffered by Tet-ON/OFF systems.

Rapid induction and depletion in a sequential manner is possible because regulation of activity occurs at the post-translational level; wherein the presence of chemical inducer becomes the limiting factor while the magnitude of supply will dictate the length of an induction effect. An important consideration with this technique is the compatibility of the protein of interest to the FKBP-DD (DD) protein tag. Because the target protein is tagged with both the DD domain (107 aa) and a fluorescent protein, the utility of this approach is limited to those proteins that can tolerate tags of this size while retaining function. The fluorescent tag can be omitted, but this will prevent intravital visualization of protein localization within the cell of interest and prevent visual confirmation of protein induction. The latter concern can be alleviated by introducing a transgene encoding a DD-tagged fluorescent protein in addition to the DD-tagged target protein. It must also be considered that in the absence of Shield-1, the target protein is translated in its entirety and may therefore be functional for a brief period prior to its degradation in the proteasome. This may also preclude studies involving proteins that affect proteasome function.

Visualizing the intracellular and biophysical impact of rapidly induced target protein offers a unique opportunity to evaluate genes of interest that have putative simultaneous effects on cell migration, morphology, and proliferation. For example, induction of E-cadherin activity within cancer xenografts resulted in the formation of junctions between mesenchymal cancer cells, causing increased cell-cell contacts within the micrometastatic colony. However, induction and subsequent depletion of a target protein may not necessarily result in a complete reversion back to the original morphology, as this will depend on the length of the time course and the nature of the effector protein. While the relevance of EMT in cancer metastasis is the focus of ongoing debate, our results demonstrate that transitions between the epithelial and mesenchymal state *in vitro* and *in vivo* can be in fact, rapid and inducible. While scores of presumed inducers of EMT have been described, the full impact of very few have been characterized as rigorously as E-cadherin. This approach can facilitate a straightforward assessment of those factors that putatively induce or revert EMT *in vivo* and to determine if these presumed factors are relevant to specific steps of cancer metastasis.

In addition to the application described herein, there are a number of complex and dynamic events, such as chemotaxis or apoptosis, that can be studied using this technique that are difficult or impractical to evaluate in other *in vivo* imaging models. For example, the “pulsed” induction of a putative chemotactic factor secreted by cancer xenografts could be used to observe the magnitude and rate of immune cell recruitment at sites of chemokine release. Similar approaches can be applied during assessment of putative pro-apoptotic factors in chemotherapy-resistant cancer cells. The analysis of apoptosis by end-point assays is limited as it relies on the absence of cancer cells at pre-determined timepoints. One can utilize this technique to quantitatively assess cancer cell death over time when target protein levels are toggled, while simultaneously providing information on the compartmentalization of the target protein prior to cell death. Overall, the optimization of several key components, including the shell-less embryo model, the development of the intravital imaging platform, and the relationship between Shield-1 dosing *in vitro* and *in vivo*, make this method highly accessible and broadly applicable to a wide range of experiments that require visualization of dynamic events *in vivo*.

## Materials and Methods

### Reagents and constructs and cells

The full length human E-cadherin cDNA clone was a kind gift of Dr. Margaret Wheelock (U of Nebraska Medical Centre, Omaha, NE) and the FKBP-DD L106P cDNA was provided by Dr. Thomas J. Wandless (Standford U, Palo Alto, CA)[12]. The TdTomato vector was a generous gift of Dr. Roger Tsien (UC San Diego) and pzsGreenC-1 vector was purchased from Clontech Inc., (Mountain View, CA). The small molecule, Shield-1, was purchased from Cheminpharma Inc, (New Haven, CT) and reconstituted in 100% EtOH. The monoclonal antibody for E-cadherin was from BD Pharmingen (Burlington, ON), the monoclonal antibody for vimentin was from Dako (clone 3B4, Burlington, ON), the monoclonal antibody to detect 20S proteasome was Subunit β1, clone MCP421 from Biomol (Burlington, ON), and the Goat anti-mouse Alexa647 secondary antibody and Hoechst 33345 were from Invitrogen (San Diego, CA). The lymphotrophic MDA-MB-231-luc-D3H2LN (231LN) cell line was from Caliper Life Sciences (Hopkinton, MA). MEM media supplemented with 10% FBS, sodium pyruvate and non-essential amino acids (NEAA) was used for cell culture (Invitrogen). To facilitate detection of the breast cancer cells *in vitro* and *in vivo*, a stable red fluorescent protein-expressing variant was made by transfection of tdTomato into 231LN cells. Briefly, 2 µg of tdTomato cDNA in pcDNA-3.1 hygro was nucleofected into cells using the Nucleofector device as specified by manufacturer (Lonza, Burlington ON, Canada). Nucleofected cells were plated in 6 well dishes for 24 hours prior to adding drug selection pressure for 14 days. Complete media containing hygromycin (700 mg/ml) was replenished every 3 days. High tdTomato expressing clones were isolated by fluorescence activated cell sorting using a BD FACSVantage DiVa cell sorter equipped with 488 nm/633 nm/UV lasers. To generate the pzsG-DD construct, the destabilization domain (DD) cDNA was inserted into the 3′ end of the zsGreen sequence in the pzsGreen-C1 with a 4 alanine residue linker. The human E-cadherin cDNA was inserted into the 5′ end of the zsGreen sequence in the pzsG-DD construct (pEcadh-zsG-DD) or into the 5′ end of the zsGreen sequence in the pzsGreen-C1 construct (pEcadh-zsG). The 231LN cell was stably transfected with a construct that constitutively expressed the red fluorescent protein tdTomato. These 231LN-tdTomato cells were then stably transfected with either pzsGreen, pzsG-DD, pEcadhzsG, or pEcadh-zsG-DD constructs.

### Fluorescence microscopy

For static confocal fluorescence imaging, an Olympus Fluoview confocal microscope was used and ImageJ was used for all image processing. For time-lapse live cell imaging, a Zeiss Axiovert upright fluorescence microscope with both 10X and LD 20X objectives and a Weatherstation to maintain temperature and humidity were used. For intravital spinning disk confocal microscopy, a specialized instrument (Quorum Technologies, Guelph, ON, Canada) comprised of an upright Zeiss AxioExaminer Z1, Ludl filter wheels and large format motorized stage, a Yokogawa spinning disk head, a Hamamatsu 9100-12 ImageEM CCD camera, controlled by Volocity (Improvision, UK) was used to acquire all images and all image processing, image analysis and movie development was done with Volocity and ImageJ [29].

### Imaging of cells in culture

For all *in vitro* experiments, stable cell lines were plated on 24 mm No. 1 circular coverslips at 70% confluency prior to Shield-1 or vehicle treatment unless stated otherwise. To fix cells, 1% paraformaldehyde in PBS pH 7.2 was used and 0.05% Saponin with 1% albumin in PBS pH 7.2 was used to permeabilize cells for immunofluorescence staining. Prolong Gold Anti-fade mounting media (Invitrogen, CA) was used to mount cells onto glass slides. For real-time live cell imaging, cells plated onto circular coverslips were transferred and mounted into a specialized imaging chamber for use with an upright microscope. This specialized imaging chamber is a closed system and was supplemented with 20 mM HEPES buffer (pH 7.2). The upright microscope is fitted with a temperature regulated enclosure to maintain a temperature of 37°C. All live cell imaging experiments were conducted for a minimum of 16 hours.

### *In vivo* videomicroscopy of avian embryo chorioallantoic membrane (CAM)

231LN cells (2×10^6^ cells/CAM) containing the zsGreen-DD or Ecadherin-zsG-DD construct were injected intravenously into a CAM venule of a day 9 old embryo as previously described in [28]. After five days of growth, Shield-1 or ethanol vehicle (in 4%EtOH, 100 µL/CAM) was injected intravenously [28], [30] to a final concentration of 1 or 2 µM Shield-1 as indicated. The average volume of a day 15 avian embryo is 8 mL[31]. Assuming Shield-1 will be uniformly distributed if injected intravenously, to establish a 1 µM final concentration of Shield-1, 100 µL of diluted Shield-1 (4 µL of 2 mM Shield-1 stock added to 96 µL PBS) was injected intravenously. To establish a 2 µM concentration of Shield-1, 8 µM of 2 mM Shield-1 stock was added to 92 µL of PBS. Vehicle injections were comprised of 100% EtOH diluted in PBS (4 µL of 100% EtOH in 96 µL of PBS for 1 µM Shield-1 injections; 8 µL of 100% EtOH in 92 µL of PBS for 2 µM Shield-1 injections). Images of multiple XY points were acquired every 15 minutes for up to 80 hours. Image acquisition parameters were optimized to visualize punctate zsGreen signal prior to Shield-1 administration, this resulted in clear visualization of E-cadh-zsG-DD adherens junctions post-Shield-1 treatment but also resulted signal saturation in later timepoints (t>12 hours).

## Supporting Information

Movie S1 ***In vitro*** **time-lapse video of 231LN cells expressing tunable zsGreen in the absence and presence of Shield-1 ligand.** Top panels represent cells treated with vehicle and the bottom panels represent cells treated with 1.0 µM Shield-1. Images were acquired every 15 minutes in the green (zsG-DD) and red (tdTomato) channels. Movie corresponds to Figure 2A, B.
(MOV)

Movie S2 ***In vitro*** **time-lapse video of 231LN cells with tunable E-cadh-zsG-DD in the absence and presence of Shield-1 ligand.** Cells were treated with vehicle (top panels) and 0.2 µM Shield-1 (bottom panels) and images were acquired every 15 minutes in the green (E-cadh-zsG-DD) and red (tdTomato) channels. Movie corresponds to Figure 5A–B.
(MOV)

Movie S3 ***In vivo*** **time-lapse video of 231LN cells expressing tunable zsG-DD in the presence and absence of Shield-1 ligand.** Images were acquired every 20 minutes in the green (E-cadh-zsG-DD) and red (tdTomato) channels. The left panel represents treatment with vehicle, the middle panel represents treatment with 0.2 µM Shield-1, and the right panel represents treatment with 0.5 µM Shield-1 treatment. Movie consists of maximum intensity projections for each timepoint and corresponds to Figure 6A–C.
(MOV)

Movie S4 ***In vivo*** **imaging of the prolonged effect of stabilized E-cadh-zsG-DD protein in 231LN cells expressing tunable E-cadh-zsG-DD with 1.0 µM Shield-1 ligand treatment.** 231LN micrometastatic colonies expressing tunable zsG-DD protein were treated with Shield-1 ligand to achieve a final *in vivo* concentration of 1.0 µM. An epithelial morphology is maintained for >40 hours. Images were acquired every 15 minutes in the green (E-cadh-zsG-DD) and red (tdTomato) channels. Movie consists of maximum intensity projections for each timepoint and corresponds to Figure 7A.
(MOV)

Movie S5 ***In vivo*** **imaging of the treatment of 231LN cells expressing tunable E-cadherin with 0.2 µM Shield-1.** No formation of E-cadherin-zsG based adherens junctions is observed using a 0.2 µM concentration of Shield-1 ligand. Movie consists of maximum intensity projections for each timepoint and corresponds to Figure 7B.
(MOV)

Movie S6 ***In vivo*** **imaging of Shield-1 mediated induction of tunable E-cadherin in 231LN cells.** 231LN micrometastatic colonies expressing tunable E-cadh-zsG-DD protein were treated with Shield-1 ligand to achieve a final *in vivo* concentration of 0.5 µM resulting in an induction effect within 24 hours of Shield-1 injection. During the induction phase, an epithelial morphology is maintained for 12 hours. Images were acquired every 15 minutes in the green (E-cadh-zsG-DD) and red (tdTomato) channels. Movie consists of maximum intensity projections for each timepoint and corresponds to Figure 7C.
(MOV)

Movie S7 ***In vivo*** **imaging of Shield-1 depleted 231LN cells expressing tunable E-cadherin.** 231LN micrometastatic colonies expressing tunable E-cadh-zsG-DD protein were treated with Shield-1 ligand to achieve a final *in vivo* concentration of 0.5 µM to induce an epithelial morphology over 12 hours (Movie S6). This movie reveals events that occur during Shield-1 depletion, such as a reversion to the mesenchymal morphology (Movie S7). Images were acquired every 15 minutes in the green (E-cadh-zsG-DD) and red (tdTomato) channels. Movie consists of maximum intensity projections for each timepoint and corresponds to Figure 7D.
(MOV)
